# Evaluating Chat Generative Pretrained Transformer (GPT-4o) Problem-Solving Performance in the Japan Certificate Examination for Biomedical Engineering Class 1

**DOI:** 10.7759/cureus.81029

**Published:** 2025-03-23

**Authors:** Kai Ishida

**Affiliations:** 1 Faculty of Engineering, Shonan Institute of Technology, Fujisawa, JPN

**Keywords:** artificial intelligence, certificate examination, chatgpt, large language models, medical engineering

## Abstract

Introduction

Chat generative pretrained transformer (ChatGPT; OpenAI, San Francisco, CA) has developed rapidly and is used in various fields, including medical engineering. Japan’s Certificate Examination for Biomedical Engineering class 1 (CEBM1) is responsible for the assessment of comprehensive specialized knowledge and skills centered on the maintenance and safety management of medical devices, systems, and related equipment. This study evaluated the performance of ChatGPT (GPT-4o) on CEBM1 for comparison to human-level expectations.

Methods

We targeted 171 questions including testing for knowledge with fundamental, applied, and problem-solving abilities from the 26th to 28th CEBM1s. We inputted the Japanese version of questions to ChatGPT (GPT-4o), and evaluated performance based on question difficulty. No prompt optimizations were used. We compared the responses provided by ChatGPT with the correct answers.

Results

The number of correct answers was 39 (68.4±10.5%) for questions testing fundamental knowledge, 33 (57.9±5.3%) for questions testing applied knowledge, and 38 (59.6±8.0%) for questions testing problem-solving ability. There was no statistically significant difference among the three groups. The passing criteria of 60% or higher was achieved only for the 28th examination. However, over 80% of the questions answered incorrectly were due to a lack of knowledge or incorrect knowledge. When asked questions about the background causes and specific countermeasures for problems related to medical devices, the questions were misunderstood, and in certain cases, answers were generated as hallucinations.

Conclusions

Currently, ChatGPT possesses a certain level of knowledge in medical engineering; however, it cannot be considered universally accurate in solving all possible problems.

## Introduction

Large language models (LLMs) have been applied across various fields. They can not only answer questions but also generate new texts, images, music, and videos. Among these models, the Chat Generative Pretrained Transformer (ChatGPT), released by OpenAI (San Francisco, CA) in 2022, is a type of LLM that has gained increasing attention for its ability to generate detailed answers to questions across various fields [1]. ChatGPT is also being used in the medical field for various purposes, such as diagnostic support for common major complaints, cancer screening, automatic generation of diagnostic reports, and medical education [2-5]. Numerous studies have examined ChatGPT’s performance on medical license examinations worldwide [6-8]. Moreover, several researchers have reported the ability of ChatGPT to pass national examinations for dentists, nurses, and other healthcare professionals [9-13]. Therefore, ChatGPT possesses fundamental clinical and medical knowledge, including pharmacy, diagnostics, and rehabilitation. However, these examinations comprise multiple-choice questions that require the examinee to select one or more optimal solutions. In addition, the national examination covers minimum medical basics and clinical knowledge necessary for certification but includes fewer questions on applied knowledge or in-depth questions. Moreover, when evaluating the accuracy of tests that test specialized knowledge, results indicate that the score for the board examinations for specialists in the Japanese Ophthalmology Society test was approximately 70% of the actual test taker's score, while its performance on the Japanese Dental Society of Anesthesiology board certification examination was below 60% [14,15]. However, as new LLM models continue to be developed, the latest version of ChatGPT may achieve greater accuracy.

Clinical engineering is a national medical qualification that requires expertise in various engineering disciplines, such as electrical, electronic, and mechanical engineering. A study evaluated ChatGPT’s accuracy in answering the Japanese National Examination for Clinical Engineers (JNECE) and reported that it was capable of passing the test, demonstrating a wealth of interdisciplinary knowledge in medicine, engineering, and clinical engineering [16]. Recently, hospitals have increasingly integrated various medical devices, electrical and medical gas facilities, and information and communication systems. Maintenance and management are important for the stable operation of these devices, facilities, and systems. In addition, in case of any issues, the ability to solve problems is essential. However, many hospitals, especially in Japan, lack clinical engineers with expertise in medical devices [17,18]. Moreover, clinical engineers do not necessarily have extensive knowledge and skills in all medical devices and related systems and facilities. With the growing application of LLMs in the medical field, it is assumed that these models have acquired a certain degree of specialized knowledge of medical engineering. However, there has been no benchmarking of their assumed knowledge.

The Japan Society for Medical and Biomedical Engineering (JSMBE) conducts the Certificate Examination for Biomedical Engineering class 1 (CEBM1), which requires additional engineering knowledge in addition to specialized medical knowledge, is conducted to assess the qualifications of individuals with comprehensive specialized knowledge and skills in the maintenance and safety management of medical devices, systems, and related equipment [19]. It also evaluates their ability to educate and instruct other medical professionals on these topics. The CEBM1 is broadly classified into three areas: medical device safety management, biological measurement, and therapeutic devices. In medical equipment safety management, the questions cover several standards related to medical devices and facilities, the application of reliability and safety engineering to medical practice, and the safety of medical materials. Biological measurements include questions on the principles and structures of various physiological function tests and diagnostic imaging devices, along with the principles and error factors of sensors. In therapeutic devices, the questions focus on the principles, structures, maintenance, and inspection of life support devices, such as defibrillators, electrocautery, blood purification systems, and ventilators. The CEBM1 comprises 19 questions structured as: three sets of three questions and one essay-type question on a certain topic for a total score of 300 points. The first set of three questions on medical engineering comprises five multiple-choice questions testing fundamental knowledge. The second set of three questions on applied knowledge comprises five multiple-choice or word/number questions. The third set of three questions tests problem-solving abilities and is a written examination. The essay-type questions require the candidate to write a few hundred words on a topic related to medical engineering. The passing rate for the last five years has remained in the 30% range, making it a challenging exam. Those eligible to take the CEBM1 are those who have passed the Certificate Examination for Biomedical Engineering class 2 (CEBM2), which is a lower qualification of the same examination, or the JNECE. Candidates who pass the CEBM1 with at least two years of practical experience in the medical engineering field are conferred the title of Certified Biomedical Engineer Class 1.

Although study has evaluated the accuracy of fundamental medical engineering, such as JNECE, studies that examine the performance of LLMs on questions involving medical engineering expertise, applied knowledge and problem-solving ability are lacking. This study aimed to evaluate the accuracy of the responses of the current ChatGPT to the CEBM1 exam to address this research gap and identify potential challenges associated with their use.

## Materials and methods

We targeted a total of 171 questions from the 26th to the 28th CEBM1 tests held between 2021 and 2023. To assess ChatGPT (GPT-4o)’s performance, we first provided the following: “Here, you undergo CEBM1. Read the question and select or write the appropriate answer. Please be careful about hallucinations.” However, no further special prompt tunings have been conducted. We input the Japanese version of each question, including images and options from the CEBM1 test. The images were scanned from the question paper at 600 dpi and entered. We compared the answer options provided by ChatGPT with the correct answers provided by JSMBE. For written questions (second and third questions), answers that matched the model answer were considered correct, while partial points were not awarded. On the other hand, answers that were essentially the same as the model answer were considered correct. For example, in response to the solving-ability question “Why is the operating time of an uninterruptible power supply regulated to be short, at least 10 minutes?”, if the model answer was “To act as a bridge until the emergency generators are operational,” and the generated answer was “Because it is intended to be used in conjunction with a private power generating facility,” it was deemed to be the correct answer. The CEBM1 has a maximum score of 300 points; however, the scoring for each question was not announced. In this study, the distribution of questions was defined as three points for the fundamental knowledge of the first question, five points for the applied knowledge of the second question, seven points for the problem-solving ability of the third question, and 15 points for the essay-type questions. As the scoring criteria for the essay-type questions were unclear, we decided only to check the generated content and compared it with the model answers without assigning a score. The generated answers were compared with the model answers from CEBM1, and obvious errors in knowledge were considered hallucinations. All input work was conducted between November and December 2024 by a researcher specializing in medical engineering with over 10 years of experience as a clinical engineer. In addition, as a quantitative evaluation, we calculated the percentage of correct answers for each of the three groups: fundamental knowledge, applied knowledge, and problem-solving ability. The statistical analysis of fundamental knowledge applied knowledge, and problem-solving ability was performed via Microsoft Excel 2021 (Microsoft, Washington, DC) using the chi-squared test. Furthermore, the reasons for incorrect answers were tabulated and categorized into lack of knowledge or incorrect knowledge, misunderstanding of the questions, calculation errors, and image recognition problems.

## Results

We present an example of certain questions and their responses by ChatGPT. While both the input text and the generated answers were written in Japanese, all content in this paper has been translated into English. The consistency between the translated English and Japanese was checked by the researcher.

**Figure 1:**
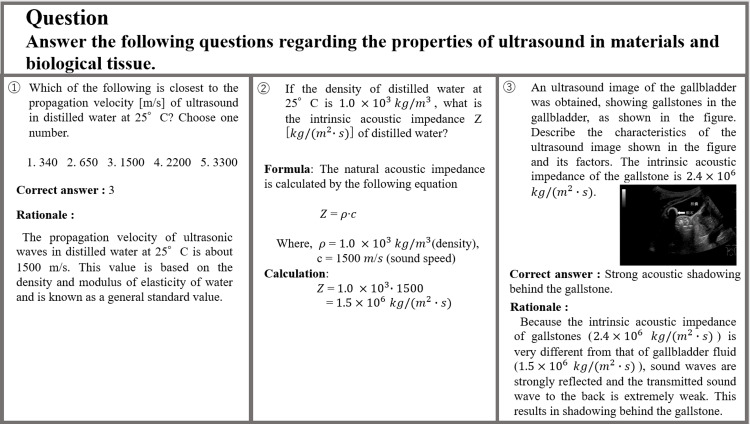
Questions on ultrasound characteristics for living organisms and ChatGPT’s response

Figure 1 shows the questions and responses related to the characteristics of ultrasound on living organisms. The first question, which asked about the propagation velocity of ultrasound in water, was answered and explained correctly. The second question, which was concerned with the intrinsic acoustic impedance under specific conditions, was correctly calculated and explained. The third question required an interpretation based on the image. ChatGPT’s response was relevant, correctly explaining that the shadow was caused by the reflection of sound waves owing to the difference in impedance between the gallstones and gallbladder fluid.

**Figure 2:**
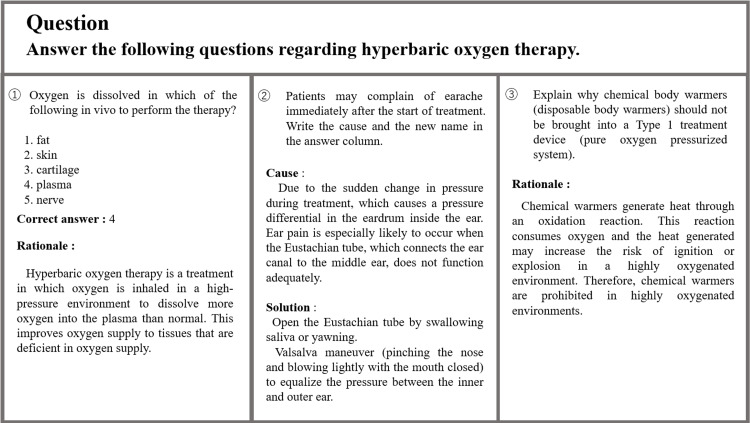
Questions on problems related to hyperbaric oxygen therapy and ChatGPT’s response

Figure 2 shows the questions and answers to the problems related to hyperbaric oxygen therapy. The first question, which asked about the principles of treatment, was answered and explained correctly. The second question was concerned with events that may occur during treatment and asked about their causes and countermeasures. The correct answer and explanation were obtained for this as well. The third question, which tested the knowledge necessary to prevent accidents and the underlying mechanism, was answered with the correct knowledge.

**Figure 3:**
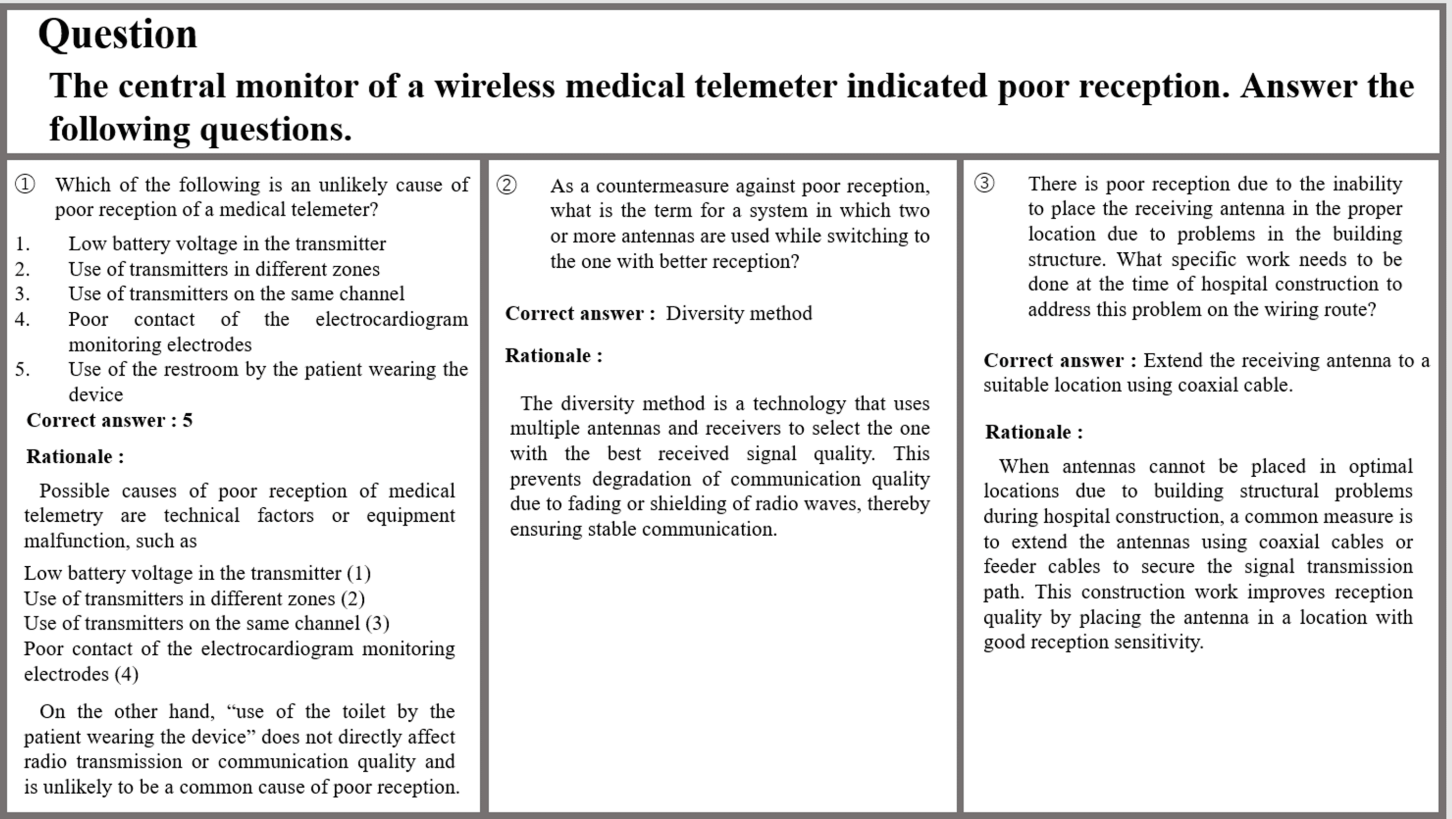
Questions on problems related to poor reception of wireless medical telemeters and ChatGPT’s response

Figure 3 shows the questions and answers about the problems related to the poor reception of the wireless medical telemeter. The first question asked the test-takers to select an unlikely cause of poor reception. Although the answer states that “Use of the restroom by the patient wearing the device” does not cause poor reception, restrooms are shielded spaces that may affect radio wave propagation and cause poor reception. Furthermore, the correct answer, “Poor contact of electrocardiogram monitor electrodes,” may not affect the electrocardiogram measurement, but it does not affect the poor reception of radio waves. Thus, this is considered a hallucination. The second question was on the antenna reception method as a countermeasure against poor reception, and the correct answer and explanation were obtained. The third question asked what measures should be taken to prevent poor reception during the construction stage of a hospital. The model answer was “Pre-drill a through hole and install a conduit in the concrete wall or beam in the ceiling that will serve as the wiring route for the receiving antenna.” The generated answer was one of the measures in improving poor reception; however, as the question asked on “work needs to be done at the time of hospital construction to address this problem on the wiring route?” extension work using cables required the securing of a route for laying the cable itself in the first place.

**Figure 4:**
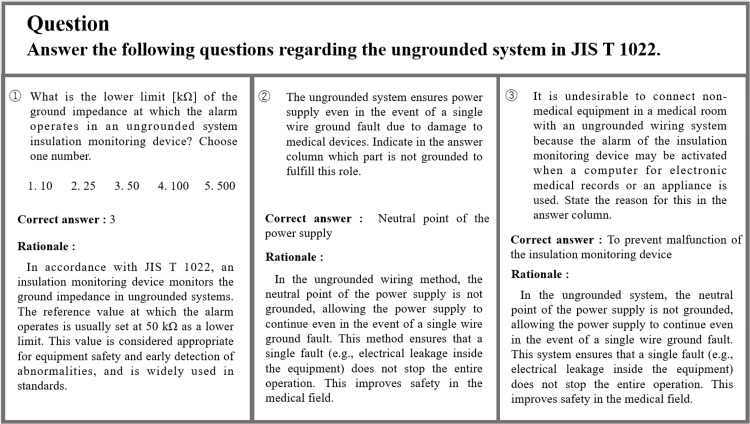
Questions on ungrounded systems specified in the JIS T 1022 and ChatGPT’s response JIS, Japanese Industrial Standards

Figure 4 shows the questions and responses on ungrounded systems specified in the Japanese Industrial Standards (JIS) T 1022, which set standards for hospital electrical equipment. The first question asked about the lower limit value of the ground impedance specified by the standard. The correct value was selected, and the explanation was also correct. The second question asked about the principle of ungrounded systems. The answer was “neutral point of the power supply”; however, the correct answer was “after the neutral point of the power supply (the secondary side of the isolation transformer),” which is essential for the realization of an ungrounded system. Although the description of the role of the ungrounded systems was accurate, it misstated all the points that favored floating. Although not a complete answer, it could be eligible for partial points. The third question was a typical case that should be noted in medical rooms with an ungrounded system and is also a problem often encountered in operating rooms. The answer stated that “to prevent malfunction of the insulation monitoring device;” however, the insulation monitoring device monitors the leakage current, and the answer was thus completely incorrect. Moreover, the explanation did not correctly explain the causes of the insulation monitoring device erroneously issuing an alarm, thereby suggesting that the question text was not properly understood.

**Figure 5:**
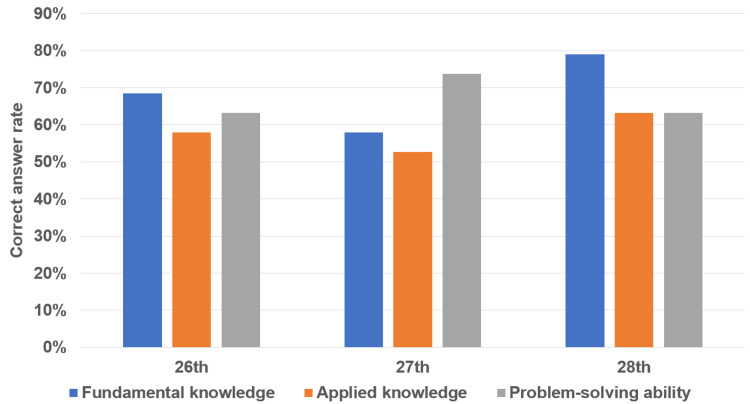
Total ChatGPT performance on the 26th to 28th CEBM1 CEBM1, Certificate Examination for Biomedical Engineering class 1

The overall results are summarized in Figure 5. The average correct answer rates were 68.4±10.5% for questions testing fundamental knowledge, 57.9±5.3% for questions testing applied knowledge, and 59.6±8.0% for questions testing problem-solving ability. However, there was no statistically significant difference among the three groups.

**Figure 6:**
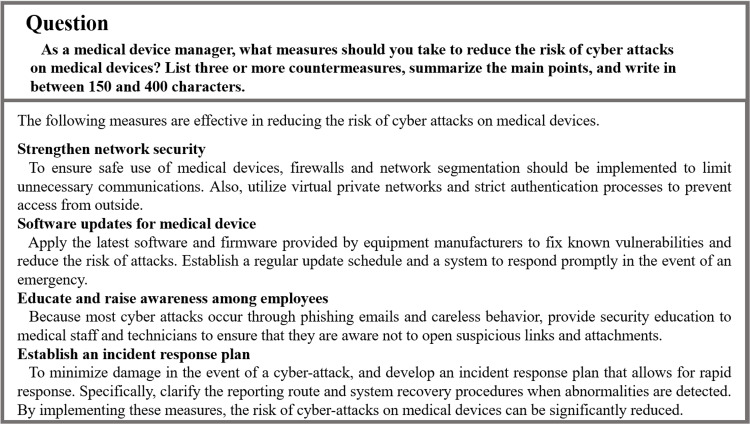
Essay-type question and ChatGPT’s response

Figure 6 shows the questions and answers to the essay-type question. The answers matched those shown in the model answers. However, for all the questions, answers with more than the specified number of characters were generated.

According to the scoring system defined in this study, the score from the ChatGPT was 169.7 ± 9.6 points, excluding the essay-type question. The examination with the highest score was the 28th CEBM1, with 180 points.

## Discussion

The questions testing fundamental knowledge assessed the knowledge required for medical engineering at the CEBM2 level, a lower qualification of CEBM1. On average, the correct answer rate for these questions exceeded 60%. Furthermore, based on previous studies, ChatGPT can successfully pass the JNECE and has a sufficient level of knowledge regarding CEBM2, which has many questions in the same field and difficulty as in this examination [16]. However, while the answer accuracy for the JNECEs was 86%, the answer accuracy for fundamental knowledge was slightly lower at approximately 60%. This is attributed to the fact that CEBM asks questions from an actual field perspective; thus, the examinations are more focused on applied ability than fundamental textbook knowledge. However, for questions that tested applied knowledge and problem-solving ability, the correct answer rate was lower than that for fundamental knowledge. Many of these questions were in written form, and certain questions generated content that caused hallucinations, leading to a low rate of correct answers. However, we confirmed that for certain answers, there was a possibility of receiving partial points even if a complete answer was not possible. In this study, we decided not to provide partial points; however, additional points may be ascribed based on actual scoring standards.

Each time the essay-type question was administered, the content was related to information security related to medical devices. For these questions, the generated answers summarized the main points, although the number of characters exceeded the prescribed number of characters. This indicated that examinees had practical knowledge in addition to general literacy. When inputting question sentences into ChatGPT, generating questions with the appropriate number of characters is possible by adjusting the prompt to adhere to the specified number of characters or by condensing excessive sentences within the specified number of characters.

The passing standard for CEBM1 has not been clearly stated but is generally considered to be 60% or higher. The passing rate of actual examinees for the 26th to 28th exams was 36.9%, 33.5%, and 35.8, respectively. Thus, the difficulty level will not change significantly. In this study, the evaluation was performed based on the scoring system defined by the author. Therefore, we discussed this while clearly stating that the actual scoring criteria may differ. According to the scoring criteria set in this study, the average overall score for the ChatGPT excluding the essay-type question was 169.7±9.6 points. Assuming a total score of 285, excluding 15 points for the essay-type question, the average score rate is 59.5%, just below 60%. However, for each examination type, the 28th examination yielded a score of 180, with a score rate of 63%, which was at the passing level. Furthermore, there were cases wherein people passed the examination with a score rate of less than 60%. Based on these results, ChatGPT (GPT4-o)'s current knowledge of medical engineering is considered as close to the passing level of CEBM1.

CEBM1 asks questions related to problems that can occur in the field using medical devices and related facilities or systems and the knowledge and skills that are important for ensuring medical safety [19]. In addition to fundamental medical and engineering knowledge, candidates must be capable of addressing various problems that may occur in the field, such as electric shocks, ground faults, noise contamination in measuring biological signals, interference with wireless medical telemeter radio wave reception in hospitals, and rapid administration of infusions owing to siphoning. Based on the results, ChatGPT has learned fundamental on-site knowledge, such as the safety management of medical devices and related equipment, principles, and structures of measurement and diagnostic equipment, various treatment devices, and precautions for use. However, when asked questions about the background causes and specific countermeasures for problems related to medical devices, the questions were misunderstood, and in certain cases, answers were generated as hallucinations. Moreover, certain problems were found to arise, including simple calculation errors and the inability to provide answers under specified conditions. This suggests that CEBM1 exceeds the textbook level and requires practical knowledge and that ChatGPT lacks learning in these areas. In a previous study, we evaluated the Healthcare Information Technologist examinations using ChatGPT as a benchmark for comprehensive knowledge about healthcare information systems, but similar to the results of this study, the accuracy was low for questions that required practical knowledge [20]. Therefore, the current ChatGPT (GPT-4o) cannot be considered fully capable of solving all-around problems related to medical engineering. The knowledge required for CEBM1 is not only textbook-based but also a lot of know-how. If ChatGPT can learn this properly, we believe it could become a useful tool in the field of medical engineering.

This study has limitations. First, this study only targeted and evaluated Japanese certification examinations. There are certifications in other countries for those responsible for the maintenance and management of medical devices, such as biomedical equipment technicians in the United States, but the questions in those examinations were not targeted. This is a benchmark for the level of questions in the Japanese CEBM1 examination. In this study, no special prompt adjustments were made to ChatGPT. ChatGPT’s output changes depending on the content of the input prompt; however, the accuracy of this improvement is small, and depending on the field, the correct answer rate may even worsen [14]. Recent advancements, such as retrieval-augmented generation (RAG), have been reported to reduce the generation of hallucinations and improve answer accuracy by allowing LLMs to refer to reference external data [21]. In this study, there is a possibility that highly accurate answers can be obtained by referring to the model answers of CEBM1 in RAG. We only used questions from a limited number of years (three years) to evaluate accuracy; however, in the future, we can expect accuracy to improve by storing more examples and exam questions in RAG. Moreover, we targeted only one LLM of ChatGPT (GPT-o) in this study. However, the performance may improve with other reasoning models, such as Open Artificial Intelligence Model Version 1 (OpenAI-o1 (OpenAI, San Francisco, CA)). It has been reported that OpenAI-o1 has higher accuracy than GPT-4o for tests that require specialized medical knowledge [22]. Therefore, we believe that by targeting these models in the future, it will be possible to evaluate problem-solving skills that require highly specialized knowledge, such as in medical engineering.

## Conclusions

In this study, we analyzed ChatGPT’s responses to CEBM1 to evaluate the applied knowledge of medical engineering and problem-solving ability using ChatGPT. With an increase in the question difficulty - from fundamental to applied and problem-solving questions - the average percentage of correct answers decreased. In addition, while certain generated answers were accurate, others misunderstood the questions, and some generated hallucinations. Thus, currently, ChatGPT possesses a certain level of knowledge in medical engineering; however, it cannot be considered universally accurate in solving all possible problems.
